# Abdominal splenosis mimicking peritoneal deposits- A case report.

**DOI:** 10.11604/pamj.2014.17.269.3413

**Published:** 2014-04-12

**Authors:** Kamini Gupta, Archana Ahluwalia, Tanica Jain, Kavita Saggar

**Affiliations:** 1 Department of Radiodiagnosis, Dayanand Medical College and Hospital, Ludhiana, India

**Keywords:** Abdominal splenosis, peritoneal deposits, splenic trauma

## Abstract

Splenosis is a benign condition among patients with a history of splenic trauma or surgery. Most cases of splenosis are intra abdominal due to direct seeding of surrounding structures, although these heterotopic rests may occur almost anywhere in the body, and its diffuse nature may raise the suspicion of metastatic cancer. The increased prevalence of abdominal trauma due to road accidents and the growing armamentarium of available imaging modalities suggest that abdominal splenosis may be expected more often than ever. We, in this article emphasize the crucial role of taking a thorough patient's medical history concerning splenic trauma in the past and the use of novel non invasive diagnostics modalities that allow accurate diagnosis.

## Introduction

Splenosis is an uncommon benign condition resulting from heterotopic auto transplantation of splenic tissues onto exposed vascularised intra and extraperitoneal surfaces following splenic trauma and surgeries [1]. It usually occurs within the abdominal and pelvic cavity, involving visceral and parietal peritoneum. Most of the patients with splenosis are asymptomatic, however occasionally, abdominal splenosis can mimic peritoneal malignancy on imaging and pose a diagnostic dilemma. We in this article emphasize that in patients with previous history of splenic trauma or surgery, clinicians must consider the existence of splenosis, and take some measures, such as scintigraphy with (99m) Tc labeled heat-denatured erythrocyte rather than biopsy, to diagnose it correctly and to prevent unnecessary operations.

## Patient and observation

**Figure 1:**
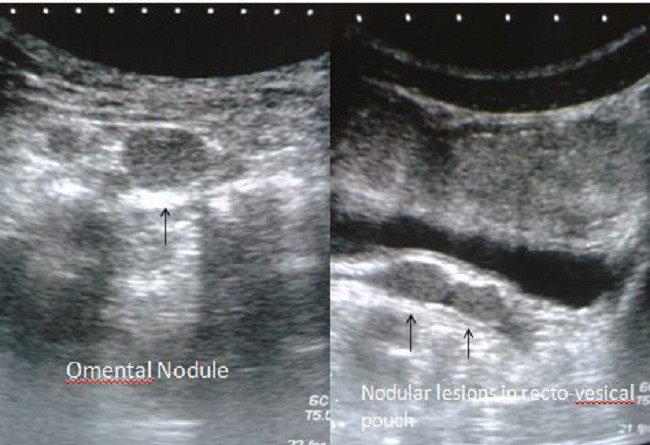
Ultrasound image in the epigastrium showing two rounded solid nodular lesions in the omentum (left) and rectovesical pouch (right)

**Figure 2:**
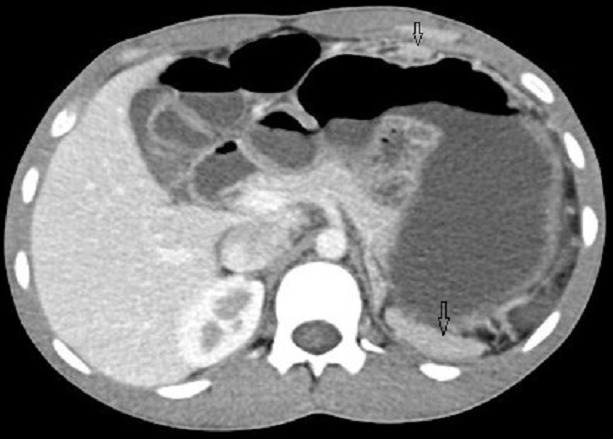
Axial image of contrast enhanced CT showing homogenously enhancing omental and left lumbar lesions (arrows)

A 23years old male patient presented to the gastroenterologist with complaints of off and on pain in his abdomen since last 6 months. Pain was not localized to one site. It varied to different sites at different times like epigastrium, midabdomen and pelvis and was mild to moderate in severity. He was diagnosed as a case of peritoneal disease on ultrasonography at some local hospital. He had past history of road side accident and splenic injury and underwent splenectomy for that. Now he was admitted with the above complaints and was referred to our department for further work-up. On ultrasonography, multiple well defined, round to oval, solid, homogenous lesions of different sizes were found in left lumbar region, in omental fat anteriorly and in the pelvis in rectovesical pouch (Figure 1). No calcification was seen in the lesions. No other positive finding was there in the abdomen. Further contrast enhanced Computed Tomography (CT) was done, which revealed homogenous significant enhancement in these lesions (Figure 2). Additional lesion was found in the root of mesentery on CT, (not shown). A provisional diagnosis of abdominal splenosis was considered in view of previous history of splenic trauma and surgery.

**Figure 3:**
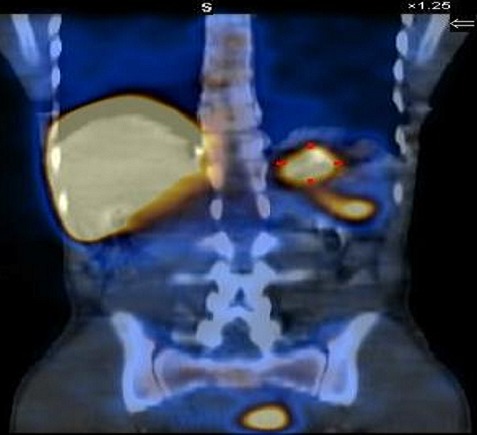
Coronal image of Tc-99m sulfur colloid test showing radionuclide uptake in the left lumbar lesions and pelvic lesions in addition to normal uptake by liver

99m Tc- labeled heat-denatured erythrocyte scintigraphy was done which confirmed the diagnosis by showing uptake in these lesions (Figure 3).

## Discussion

Splenosis means that spleen fragment implants into other sites randomly, not spleen bed, to row and form small spleens which have the physical function similar to normal spleen. It can eliminate the aged blood cells and maintain normal immunological function which is very important to attack infection [2].

The heterotopic implantation of splenic tissue after splenic trauma or splenectomy is an under diagnosed entity, occurring in up to 67% of patients who have suffered from a splenic rupture. The true incidence of this rare condition is unknown, because splenosis is usually an incidental finding at imaging or surgery. The implants can be solitary or multiple, and can occur throughout the peritoneal cavity or chest [3].

Although splenic implants are generally asymptomatic, they can lead to recurrent episodes of abdominal pain, as in our case or small bowel obstruction secondary to adhesive bands of splenic implants. Occasionally, it can cause gastrointestinal bleeding, intraperitoneal nodule infarction, hematoma, enlarging abdominal or pelvic masses, ureteral compression and hydronephrosis or recurrence of hematologic diseases treated with splenectomy [4].

A definite preoperative diagnosis of splenosis requires a high index of suspicion. A detailed medical history, thorough physical examination, lack of typical changes in the blood smear often present after splenectomy (Howell-Jolly bodies, reticulocytosis), and protective levels of antipneumococcal antibodies in a non-vaccinated patient should make the physician consider this rare condition.

Widely available imaging modalities like abdominal ultrasound examination, contrast enhanced CT scan and magnetic resonance imaging (MRI) are of limited value in the diagnostic management of abdominal splenosis. Imaging findings are not specific in this entity and reveal round and oval soft-tissue masses in the various abdominal locations. On contrast enhanced CT they have density and enhancing characteristics similar to expected density of the spleen in a splenectomized patient.

Therefore nonspecific characteristics of splenosis on imaging may be confused with numerous conditions such as metastatic disease, abdominal lymphoma, hemangiomatosis, peritonel mesothelioma, multifocal endometriosis, primary renal or hepatic malignancy, gliomatosis peritonei, granulomatous peritonitis as the consequence of disseminated infection such as tuberculosis or histioplasmosis, rupture of the tumor or a hollow viscus, or, simply, reactive adenopathy.

The current diagnostic modality of choice for splenosis is noninvasive nuclear scintigraphy. The Tc-99m sulfur colloid test of the liver and spleen was first used to diagnose splenosis due to the ability of the radio-labeled colloid to localize in the reticuloendothelial system. However, scintigraphy using Technetium-99m heat-damaged erythrocytes (RBC) or Indium 111-labeled platelets is more sensitive and specific for splenic uptake, making these tests the current diagnostic tools of choice [5, 6].

Recent case reports have shown ferumoxides-enhanced MRI as a novel technique for diagnosing splenosis. Ferumoxides are superparamagnetic iron oxides that are removed from circulation by the reticuloendothelial system [7].

Once splenosis is confirmed, no further workup is necessary unless the patient is symptomatic. No cases of death secondary to splenosis have been reported. Splenosis must be differentiated from accessory spleens. Apart from the role of thorough history, inherent differences between the two entities are quite distinct. Accessory spleens are congenital and arise from the left side of the dorsal mesogastrium during the embryological period of development. Accessory spleens are usually located near the splenopancreatic or gastrosplenic ligament. Even when they have been reported within the pancreas, kidney, scrotum and as an adenexal mass, they always conform to their embryological geographic limits. Splenosis, on the other hand, is an acquired condition and splenosis tissue can be found in any intraperitoneal or extraperitoneal location. The most common areas are the peritoneum, omentum and mesentery. However, splenosis has also been reported in the pericardium, subcutaneous tissue and even in the occipital pole of the brain. Accessory spleens are usually few in number, totaling six or less. On the other hand, 100 or more individual splenic nodules are commonly found in splenosis and greater than 400 have been reported. An accessory spleen has normal splenic histology with its blood supply uniformly arising from a branch of the splenic artery. The blood supply in splenosis however, is derived from the surrounding tissues and vessels, without any association to the splenic artery. The histological findings of the two also vary.

## Conclusion

Splenosis should be included in the differential diagnosis in all patients with abdominal, pelvic, thoracic or subcutaneous nodules with a history of splenic trauma or spleen removal. This history is paramount and should immediately broaden a clinician's differential. Once considered, the diagnostic workup for this mostly benign condition is simple, inexpensive, noninvasive, and may prevent future stress and procedures.
